# Supplementary material for: Implementing Pharmacogenomics Testing: Single Center Experience at Arkansas Children’s Hospital
Source: J Pers Med. 2021 May 11;11(5):394. doi: 10.3390/jpm11050394 (PMC8150685; doi:10.3390/jpm11050394)
Supplement: Supplementary file 1 [file jpm-11-00394-s001.zip › jpm-1187278-supplementary file.pdf]

Journal of *Personalized Medicine*

MDPI

Implementing Pharmacogenomics Testing: Single Center Experience at Arkansas Children's Hospital

Pritmohinder S. Gill [1,2,*,†], Feliciano B. Yu, Jr [3,†], Patricia A. Porter-Gill [2], Bobby L. Boyanton, Jr [4], Judy C. Allen [5], Jason E. Farrar [1,2,6], Aravindhan Veerapandiyan [2,7], Parthak Prodhan [2,8], Kevin J. Bielamowicz [1,2,6], Elizabeth Sellars [1,2,9], Andrew Burrow [1,2,9], Joshua L. Kennedy [1,2], Jeffery L. Clothier [10], David L. Becton [1,2,6], Don Rule [11] and G. Bradley Schaefer [1,2,12]

1 Department of Pediatrics, University of Arkansas for Medical Sciences, Little Rock, AR 72202, USA; JEFarrar@uams.edu (J.E.F.); KJBielamowicz2@uams.edu (K.J.B.); EASellars@uams.edu (E.S.); TABurrow@uams.edu (A.B.); KennedyJoshuaL@uams.edu (J.L.K.); BectonDavidL@uams.edu (D.L.B.); SchaeferGB@uams.edu (G.B.S.)
2 Arkansas Children's Research Institute, Little Rock, AR 72202, USA; PortergillPA@archildrens.org (P.A.P.-G.); AVeerapandiyan@uams.edu (A.V.); ProdhanParthak@uams.edu (P.P.)
3 Pediatrics and Biomedical Informatics, University of Arkansas for Medical Sciences, Little Rock, AR 72202, USA; Pele.Yu@archildrens.org
4 Departments of Pathology and Laboratory Medicine, University of Arkansas for Medical Sciences and Arkansas Children's Hospital, Little Rock, AR 72202, USA; bboyanton@uams.edu
5 Informatics, Arkansas Children's Hospital, 1 Children's Way #512-10, Little Rock, AR 72202, USA; AllenJC@archildrens.org
6 Pediatric Hematology/Oncology, Arkansas Children's Hospital, 1 Children's Way #512-10, Little Rock, AR 72202, USA
7 Pediatric Neurology, Arkansas Children's Hospital, 1 Children's Way, Little Rock, AR 72202, USA
8 Pediatric Cardiology, Arkansas Children's Hospital, Little Rock, AR 72202, USA
9 Section of Genetics and Metabolism, Arkansas Children's Hospital, Little Rock, AR 72202, USA
10 Psychiatry, College of Medicine, University of Arkansas for Medical Sciences, Little Rock, AR 72205, USA; JLClothier@uams.edu
11 Translational Software, Inc.,TSI, Bellevue, WA 98005, USA; Don.rule@translationalsoftware.com
12 Arkansas Children's Hospital NW, Springdale, AR 72762, USA
* Correspondence: PSGill@uams.edu; Tel.: +1-(501)-364-1418; Fax: +1-(501)-364-3654
† Authors contributed equally.

Copyright: © 2021 by the authors. Submitted for possible open access publication under the terms and conditions of the Creative Commons Attribution (CC BY) license (http://creativecommons.org/licenses/by/4.0/).

Supplementary Materials:

The following supplementary materials are available online at https://www.mdpi.com--------, Figure S1-S2: Comprehensive pharmacogenomic report for a mock patient shown here capture few highlights of the report and is presented in Supplementary Figure S1 and Supplementary Figure S2

Arkansas Children's
HOSPITALS • RESEARCH • FOUNDATION

Molecular Genetic Pathology Laboratory
501-364-4245 1 Children's Way,
www.archildrens.org Little Rock, AR 72202

PATIENT INFORMATION		SPECIMEN DETAILS		ORDERED BY
**NAME:**	Test Test	**SPECIMEN TYPE:**	DNA	**L. MD**
**ACC #:**	TestPatient	**COLLECTION DATE:**	9/16/2020	
**DOB:**	10/8/2005	**RECEIVED DATE:**	9/16/2020	
**SEX:**	Female	**REPORT DATE:**	10/8/2020	

Comprehensive Pharmacogenetic Report

Current Patient Medications

Codeine, Clopidogrel

Pharmacogenetic Interactions

NEONATAL PERIOD	INFANCY	EARLY CHILDHOOD	LATE CHILDHOOD	ADULTHOOD
BIRTH - 4 WEEKS	5 WEEKS - 1 Y	2Y - 5Y	6Y - 17Y	>=18Y

CLOPIDOGREL *Plavix®*

Significantly Reduced Response to Clopidogrel (CYP2C19: Poor Metabolizer) ACTIONABLE

ADULT Consider alternative therapy. Examples of alternative drugs: prasugrel (contraindicated in TIA/Stroke patients), ticagrelor, aspirin, aspirin plus dipyridamole.

PEDIATRIC The pharmacogenetic recommendations for clopidogrel based on CYP2C19 genotypes in adults may be used with caution in children and adolescents and should be accompanied by close monitoring and testing of platelet function.

**AGE**	Neonatal Period	Infancy	Early Childhood	Late Childhood	Adulthood
**SUITABILITY**	Not Used	Caution: See Pediatric Guidelines	Caution: See Pediatric Guidelines	Caution: See Pediatric Guidelines	See Adult Guidelines

Scott SA, Sangkuhl K, Stein CM, Hulot JS, Mega JL, Roden DM, Klein TE, Sabatine MS, Johnson JA, Shuldiner AR, . Clinical Pharmacogenetics Implementation Consortium guidelines for CYP2C19 genotype and clopidogrel therapy: 2013 update. Clin Pharmacol Ther 2013 Sep;94(3):317-23.

CODEINE *Codeine; Fioricet® with Codeine*

Increased Response to Codeine (CYP2D6: Ultra-Rapid Metabolizer) ACTIONABLE

ADULT Codeine is converted into its active metabolite morphine by CYP2D6. Since this patient is a ultra-rapid metabolizer, greatly increased morphine levels are expected, and the patient is at high risk of toxicity when taking codeine. The ultra-rapid conversion of codeine to morphine in breast feeding mothers can result in high and unsafe levels of morphine in the breast milk potentially causing life threatening respiratory depression in the breastfed infant. Avoid prescribing codeine, and consider an alternative opioid or a non-opioid analgesic such as a NSAID or a COX-2 inhibitor. Unless contraindicated, available alternative opioids not sensitive to CYP2D6 function include: fentanyl, morphine, hydromorphone, oxymorphone, and tapentadol.

PEDIATRIC The pharmacogenetic recommendations for codeine based on CYP2D6 genotypes in adults are suitable for children (12 years and older) and adolescents. Caution: Regardless of their genotype, children ages 12 to 18 who are obese or have obstructive sleep apnea or a weakened respiratory system should not be prescribed codeine. Prescription cough and cold medicines containing codeine are not indicated for use in children, and their use in this age group is not recommended. Warning: Breastfeeding is not recommended when taking codeine due to the risk of serious adverse reactions in breastfed infants.

**AGE**	Neonatal Period	Infancy	Early Childhood	Late Childhood	Adulthood
**SUITABILITY**	Not Used	Not Used	Not Used	Caution: See Pediatric Guidelines	See Adult Guidelines

Crews KR, Gaedigk A, Dunnenberger HM, Leeder JS, Klein TE, Caudle KE, Haidar CE, Shen DD, Callaghan JT, Sadhasivam S, Prows CA, Kharasch ED, Skaar TC, . Clinical Pharmacogenetics Implementation Consortium guidelines for cytochrome P450 2D6 genotype and codeine therapy: 2014 update. Clin Pharmacol Ther 2014 Apr;95(4):376-82.

Drug-Drug Interactions

Powered By Translational software

Genetic Test Results For **Test Test**
Molecular Genetic Pathology Laboratory | Lab Director: Bobby Boyanton, MD, Pathologist-in-Chief | CLIA: 04D0466889

Page 1 of 38

Arkansas Children's
HOSPITALS · RESEARCH · FOUNDATION

Molecular Genetic Pathology Laboratory
501-364-4245 1 Children's Way,
www.archildrens.org Little Rock, AR 72202

PATIENT INFORMATION		SPECIMEN DETAILS		ORDERED BY
**NAME:**	Test Test	**SPECIMEN TYPE:**	DNA	L. MD
**ACC #:**	TestPatient	**COLLECTION DATE:**	9/16/2020	
**DOB:**	10/8/2005	**RECEIVED DATE:**	9/16/2020	
**SEX:**	Female	**REPORT DATE:**	10/8/2020	

No drug-drug interactions can be provided within the scope of this report at this time. Either no current medications were provided for the patient or no significant risk of drug-drug interaction is present for the patient's current medications. It is highly recommended to reanalyze the risk of drug-drug interaction if the patient is prescribed a new drug.

Unrecognized Medications: *None*

Highly elevated risk for indicated condition or adverse drug reaction. Medication can be prescribed with monitoring; alternative therapy may be needed.

Moderately elevated risk for indicated condition or adverse drug reaction. Medication can be prescribed with monitoring; therapy adjustment may be needed.

Typical risk for indicated condition or adverse drug reaction. Medication can be prescribed according to standard dosing guidelines.

PHARMACOGENETIC RESULTS

DRUG-DRUG INTERACTIONS

ACTIONABLE Recommendations are suitable for implementation in a clinical setting. Recommendations extracted from evidence based guidelines issued by international pharmacogenetic consortia, professional societies or regulatory bodies (CPIC, DPWG, FDA, EMA, CPNDA, ACMG).

INFORMATIVE Recommendations are informative and implementation in a clinical setting is optional. The evidence documenting these drug-gene associations may be limited or insufficient and may require further investigation. There are no established evidence based guidelines issued by international pharmacogenetic consortia, professional societies or regulatory bodies.

MODERATE Drug interactions of moderate severity. The clinician should assess the patient's characteristics and take action as needed.

SERIOUS Severe drug interaction or contraindicated drug combination which may produces serious consequences in most patients. This drug combination generally should not be dispensed or administered to the same patient. Action is required to reduce risk of severe adverse interaction.

Powered By Translational software

Genetic Test Results For **Test Test**
Molecular Genetic Pathology Laboratory | Lab Director: Bobby Boyanton, MD, Pathologist-in-Chief | CLIA: 04D0466889

Page 2 of 38

Supplementary Figure S1: Mock comprehensive PGx report shows patient on drugs Clopidogrel and Codeine. This lists patient genotype and phenotype status.

Arkansas Children's
HOSPITALS · RESEARCH · FOUNDATION

Molecular Genetic Pathology Laboratory
501-364-4245 1 Children's Way,
www.archildrens.org Little Rock, AR 72202

PATIENT INFORMATION		SPECIMEN DETAILS		ORDERED BY
**NAME:**	Test Test	**SPECIMEN TYPE:**	DNA	L. MD
**ACC #:**	TestPatient	**COLLECTION DATE:**	9/16/2020	
**DOB:**	10/8/2005	**RECEIVED DATE:**	9/16/2020	
**SEX:**	Female	**REPORT DATE:**	10/8/2020	

Test Details

Gene	Genotype	Phenotype	Alleles Tested
ACYP2	rs1872328 G/G	Homozygous for rs1872328 G allele	rs1872328
CACNA1S	No Pathogenic Variant Detected	Uncertain Susceptibility to Malignant Hyperthermia	See Variant Results section for this gene.
CEP72	rs924607 C/T	Altered CEP72 expression	rs924607
CYP2C	g.96405502G>A G/G	Low Sensitivity	g.96405502G>A
CYP2C19	*35/*35	Poor Metabolizer	*2, *3, *4A, *4B, *5, *6, *7, *8, *9, *10, *11, *17, *35
CYP2C9	*1/*1	Normal Metabolizer	*2, *3, *4, *5, *6, *8, *9, *11, *12, *13, *14, *15, *18, *27
CYP2D6	*1/*1 XN	Ultra-Rapid Metabolizer	*2, *3, *4, *4M, *6, *7, *8, *9, *10, *11, *12, *114, *14, *15, *17, *18, *19, *21, *29, *31, *33, *35, *38, *40, *41, *42, *43, *44, *45, *46, *47, *49, *51, *53, *54, *56A, *56B, *62, *84, *100, *101, *5 (gene deletion), XN (gene duplication)
CYP3A4	*1/*1	Normal Metabolizer	*2, *3, *12, *17, *22
CYP3A5	*3/*3	Poor Metabolizer	*3, *6, *7
CYP4F2	c.1297G>A A/A	Reduced Activity	c.1297G>A
DPYD	Activity Score: 2	Normal Metabolizer	85T>C, 703C>T, 2657G>A, 2983G>T, 1905+1G>A, 302delTinsTCAT, 1679T>G, 2846A>T, 1627T>C, 1003C>A, 557A>G, 496A>G, , *, 1601C>T, 1236G>A, c.1129-5923C>G, g.476001C>T, , c.61C>T, c.62G>A
F2<br>F5	rs1799963 GG<br>rs6025 CC	Normal Risk of Thrombosis	rs1799963, rs6025
G6PD	B/B	Normal Function	A, A-(202), A-(680), A-(968), Asahi, Canton, Chatham, Cosenza, Mediterranean, Mexico City, Orissa, Kalyan-Kerala
NUDT15	*1/*1	Normal Metabolizer	*2, *3, *5, *7
RARG	rs2229774 C/C	Normal Function	rs2229774
RYR1	No Pathogenic Variant Detected	Uncertain Susceptibility to Malignant Hyperthermia	See Variant Results section for this gene.
SLC28A3	rs7853758 C/T	Decreased Function	rs7853758
SLCO1B1	521T>C T/T	Normal Function	521T>C
TPMT	*1/*1	Normal Metabolizer	*2, *3A, *3B, *3C, *4, *8
UGT1A1	*1/*1	Normal Metabolizer	*6, *27, *80
UGT1A6	rs17863783 G/G	Normal Metabolizer	rs17863783
VKORC1	-1639G>A G/G	Low Warfarin Sensitivity	-1639G>A

Powered By Translational software

Genetic Test Results For **Test Test**
Molecular Genetic Pathology Laboratory Lab Director: Bobby Boyanton, MD, Pathologist-in-Chief CLIA: 04D0466889
Page 36 of 38

Supplementary Figure S2: PGx test details provide information on gene, genotype, phenotype, and alleles tested.
